# Organoids in the oral and maxillofacial region: present and future

**DOI:** 10.1038/s41368-024-00324-w

**Published:** 2024-11-01

**Authors:** Yufei Wu, Xiang Li, Hanzhe Liu, Xiao Yang, Rui Li, Hui Zhao, Zhengjun Shang

**Affiliations:** 1 State Key Laboratory of Oral & Maxillofacial Reconstruction and Regeneration, Key Laboratory of Oral Biomedicine Ministry of Education, Hubei Key Laboratory of Stomatology, School & Hospital of Stomatology, Wuhan University, Wuhan, China; 2 Department of Oral and Maxillofacial-Head and Neck Oncology, School of Stomatology–Hospital of Stomatology, Wuhan University, Wuhan, China

**Keywords:** Cancer models, Regeneration, Tissue engineering

## Abstract

The oral and maxillofacial region comprises a variety of organs made up of multiple soft and hard tissue, which are anatomically vulnerable to the pathogenic factors of trauma, inflammation, and cancer. The studies of this intricate entity have been long-termly challenged by a lack of versatile preclinical models. Recently, the advancements in the organoid industry have provided novel strategies to break through this dilemma. Here, we summarize the existing biological and engineering approaches that were employed to generate oral and maxillofacial organoids. Then, we detail the use of modified co-culture methods, such as cell cluster co-inoculation and air-liquid interface culture technology to reconstitute the vascular network and immune microenvironment in assembled organoids. We further retrospect the existing oral and maxillofacial assembled organoids and their potential to recapitulate the homeostasis in parental tissues such as tooth, salivary gland, and mucosa. Finally, we discuss how the next-generation organoids may benefit to regenerative and precision medicine for treatment of oral-maxillofacial illness.

## Introduction

Due to the specificity of anatomy and unhealth lifestyle, oral and maxillofacial region (OMR) is highly vulnerable to a wide spectrum of diseases including inflammation,^1^ trauma,^2^ and tumors.^3,4^ Treatments of OMR illnesses are difficult in that the excision of the lesion can lead to the destruction of the OMR structure, which is essential for basic physical functions such as chewing and swallowing. On the other hand, the inability to fix up OMR with expected esthetics feature may cause severe but overlooked psychological damage to patients. Except for surgery, the development of regenerative and precision medicine has provided novel strategies and/or conceptions to manage patients with oral and maxillofacial diseases. However, the studies of this intricate entity are always challenged by a lack of versatile preclinical models.

Organoids are three-dimensional (3D) culture outgrowth derived from pluripotent stem cells (PSCs) or adult tissue stem cells (ACSs). Studies have reported that organoids can faithfully recapitulate the genotype and histological features of parental tissues. Co-culture system of organoid with stromal cells like fibroblasts, endothelial cells and immune cells, which is also called the assembled organoid, provide a novel platform to investigate cellular crosstalk in physiological and pathophysiological environments.^5–7^ Engraftment of organoid in vivo accelerate the healing efficiency of tissue including mucosa and bone.^6,8^ Drug screening based on patient-derived tumor organoids (PDOs) possesses higher clinical relevance and predictive accuracy compared to that on traditional 2D cell lines and xenograft models.^9–11^ Therefore, it is believed that the organoid industry has pushed forward the progression of regenerative and precision medicine.

Previously, Gao et al. have summarized the exiting literature refer to oral organoids, including tooth germ, salivary gland, lingual epithelium and taste bud.^11^ In this review, we further retrospect the construction of comprehensive oral and maxillofacial organoid culture system, focus on constructing, identifying, and editing organoids. Modified bioengineering approaches were summarized to generate assembled oral and maxillofacial organoids. Finally, based on this technical summary, we discuss the challenges and solution strategies for constructing assembled organoids, envisioning their therapeutic prospects and clinical application.

## Construction and characterization of oral and maxillofacial organoids

### Three key elements to construct oral and maxillofacial organoids

**Fig. 1:**
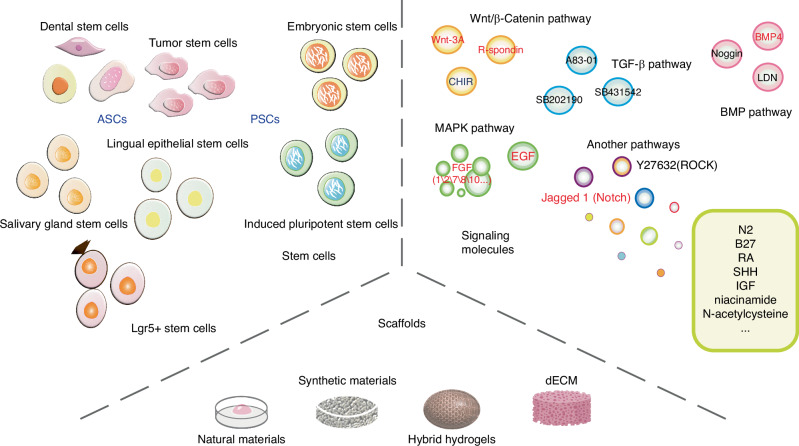
Three key elements to construct oral and maxillofacial organoids. Stem cells, scaffolds and signaling molecules are the three key elements for generating oral and maxillofacial organoids. ASCs, adult stem cells; PSCs, pluripotent stem cells; dECM, decellularized extracellular matrix; Due to the large number of signaling molecules, the full name are seen in the ‘signaling molecules’ section of the text, which will not be repeated here. The black word indicates inhibition, and the red word indicates activation

Oral and maxillofacial organoids can be generated using either ACSs or PSCs from mice and human. The maintenance of organoids growth and differentiation is largely affected by the supporting scaffolds and supplement molecules that model the environmental cues in oral and maxillofacial tissues (Fig. 1). Specifically, the generation of organoids from several tissues, including tooth,^12,13^ salivary gland (SG),^14^ tongue^15^ and mucosa,^16^ have already been summarized in previous literature. Whereas organoids of other tissues such as bone, as well as that of multiple inflammatory, premalignant, and malignant diseases remain further investigated. In any case, the *source of stem cells*, *supporting scaffolds* and *supplements molecules* are key bioengineering elements to construct oral and maxillofacial organoids.

#### Stem cells

ASCs or PSCs exhibit the capability of self-renewal and multipotent differentiation, allowing for their expansion and further induction into a variety of specific cell lineages under appropriate conditions. Organoids can mimic the complete differentiation lineages of progenitor tissues, which is crucial for their potential as disease models. Various ASCs derived from oral and maxillofacial organs have been successfully utilized in the construction of related organoids, such as dental epithelial (DE) and dental mesenchymal (DM) cells from tooth germ,^17^ dental pulp stem cells (DPSCs),^12^ periodontal ligament stem/progenitor cells (PDLSCs),^18^ SG stem cells,^19^ Lgr5+ taste stem cells,^20^ and lingual epithelial stem cells (LESCs).^15^ For instance, Calabrese et al. demonstrated the potential of DPSCs and PDLSCs to self-assemble into dentin-pulp and PDL-cementum organoids, confirming the potential of these two cell types to form fully developed tooth-like structures.^18^ Endeavors have also been made to construct tumor organoids, such as those for oral cancer and salivary gland tumors, using cancer stem cells (CSCs).^21,22^ So far, organoids derived from human PSCs have been extensively contributed to the construction of intestinal, retinal, cerebral, and renal organoids, but less research has been conducted to generate oral organoids. PSCs encompass embryonic stem cells (ESCs) and induced pluripotent stem cells (iPSCs). ESCs can differentiate into oral ectoderm, cultured with FGF7 and FGF10 to generate salivary gland rudiment.^23^ Recent studies have highlighted the successful differentiation of human iPSCs into tooth and osseous organoids.^24–26^

#### Supporting scaffolds

The ECM is one of the core components of tissues and organs in oral cavity. It provides structural support and mediates instructive signaling for the growth and differentiation of stem cells.^27^ Natural hydrogels, like Matrigel, mimic ECM but pose challenges in culture control due to complexity. While other natural hydrogels, such as collagen gels, have been employed in the construction of oral and maxillofacial organoids, Matrigel remains the predominant choice in this context.^28,29^ Additionally, the limitations of natural scaffolds in organoid culture extend to insufficient mechanical stiffness and low resistance to physical condition changes, restricting their widespread application. Synthetic scaffolds, e.g., polyethylene glycol (PEG), poly(ε-caprolactone) (PCL) and poly(lactic-co-glycolic acid) (PLGA), offer superior mechanical properties and are common in oral and maxillofacial organoid engineering.^30–32^ Fischebach et al., for instance, designed a PLGA-based polymer scaffold to mimic oral squamous cell carcinoma (OSCC). The platform based on PLGA scaffold replicated characteristics more akin to in vivo tissues, such as hypoxia, growth profiles, and expression of pro-angiogenic factors (e.g., VEGF, bFGF, and IL-8), than did Matrigel-based and 2D monolayer cultures.^33^ On the other hand, naturally derived polymers including gelatin, hyaluronic acid (HA), chitosan, and alginate, exhibit biocompatibility and structural similarities to natural ECM, are also commonly used matrix materials for organoid culture.

Hybrid hydrogels blend natural and synthetic materials, promising to overcome single-material limitations and allowing hybridization between natural-to-natural, synthetic-to-synthetic, and natural-to-synthetic hydrogels. Among the existing hybrid hydrogels, natural-to-synthetic hydrogels are considered a preferable choice in organoid culture, effectively integrating the advantages of both natural and synthetic materials. A 3D hydrogel culture system based on HA and containing PEG has been reported, promoting organized growth and differentiation of salivary acinar-like cells into functional acinar-like structures.^34^ Based on a series of micropores, a 3D culture system has been used for culturing salivary gland organoids, comprising a PCL nanofiber bottom and a PEG hydrogel wall. SG cells grown on the system exhibited spherical acinar-like 3D assemblies with increased structural integrity and secretory function.^31^ A 3D electrospun PLGA/PCL scaffold has been employed in tooth tissue regeneration engineering to establish appropriate DE-DM cell interactions, with the incorporation of nano-hydroxyapatite enhancing dental cell differentiation.^35^

Due to the potential immunogenicity, the utilization of Matrigel originated from mouse sarcoma cells has been impeded in human clinical transplantation.^36^ Decellularized ECM (dECM) provides an alternative, with recent advances in dECM-based biomaterials showing therapeutic potential. However, the decellularization methods employed vary depending on the target tissue and lack easy generalizability. During the decellularization process, cells and immunogenic molecules are extensively removed, while the majority of structural proteins (such as collagen, fibronectin, etc.) and macromolecules are largely preserved.^37^ Recent research efforts have expanded from direct use of dECM to the production of biomaterials from pulverized dECM powders or particles, subsequently reconstructed into various forms like hydrogels, electrospun scaffolds, and bioprinted scaffolds.^38,39^ Coppi and colleagues developed a dECM-based hydrogel system that complies with good manufacturing practices regulations and can form and develop human embryonic-derived organoids.^40,41^ To create clinically applicable dECM hydrogels, decellularized porcine small intestine mucosa/submucosa was treated via freeze-drying, milling, γ-irradiation, and pepsin/hydrochloric acid digestion. A previous study comparing the protein composition between dECM and Matrigel samples, respectively identified an average of 956 and 155 proteins, with only 91 proteins shared.^42^ Proteomic heterogeneity within and among samples was demonstrated by the observation of 1637 distinct proteins in different Matrigel samples, indicating the complexity and variability of Matrigel components. To ensure consistency and reproducibility of organoid culture outcomes, the matrix should possess defined compositions for widespread clinical application. Therefore, expanding the biological and mechanical properties of dECM to overcome limitations of its natural characteristics and broadening its application scope is crucial. Introducing various natural or synthetic materials is also essential to ensure diversity in properties and adaptability to the construction and growth of different organoid types.

#### Supplement molecules

**Fig. 2:**
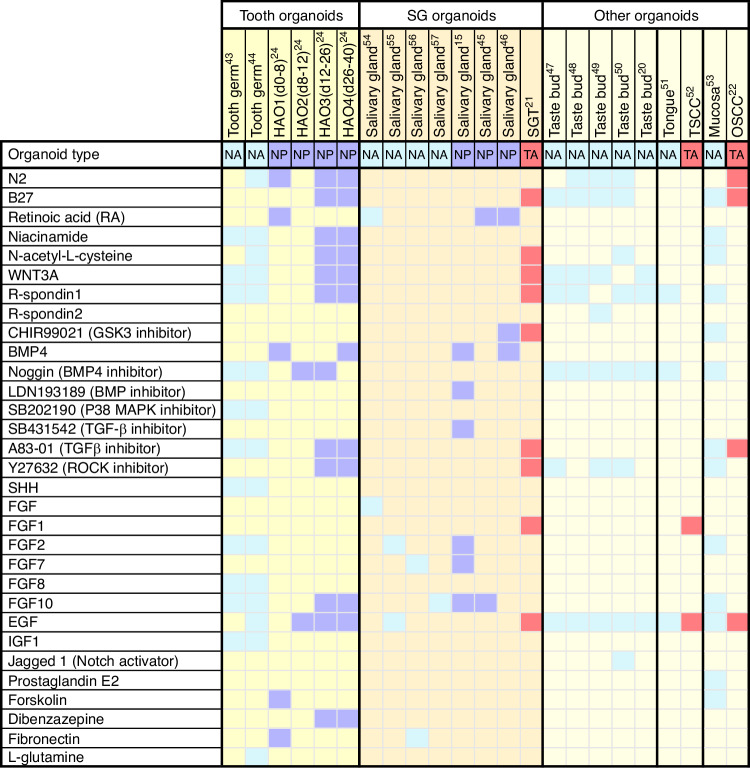
Heatmap showing the culture conditions for various oral and maxillofacial organoid cultures. Each column shows a culture condition protocol that has been reported for a particular type of organoid. Colored boxes signify the indicated growth factor component used in organoid expansion protocols. The organoid types were divided into NA, normal tissue ASC-derived organoids, NP, normal tissue PSC-derived organoids, and TA, tumor ASC-derived organoids. hAO1-4, different culture stages of the human ameloblast organoids; SGT, salivary gland tumor; TSCC, tongue squamous cell carcinoma; OSCC, oral squamous cell carcinoma

The existing culture protocols of oral and maxillofacial organoids have been summarized in Fig. 2. Of note, supplement of molecules to generate oral and maxillofacial organoids display context-dependent manner. Take tooth organoids as a case, although several factors related to WNT, EGF and FGF are employed to induce the maturation of ASC-derived tooth germ organoids, a withdrawal of these factors can otherwise promote the self-renewal of PSC at early stage when culture ameloblast organoids.^24,43,44^ On the other hand, organoids from diverse tissues may exhibit different requirement of niche factors.^20,45–53^ For instance, the WNT pathway-related proteins, such as WNT3A or Rspondins, are essential for tooth and mucosa organoids but not for salivary gland organoids^54–57^ (Fig. 2). The mucosa tumor organoids have a decline demand for supplements when compared to normal mucosa,^22,58,59^ while the salivary gland tumor organoids are more susceptible to the niche factors than their normal counterpart.^21^ The source of stem cells may also affect the selection of supplement molecules. In both tooth and salivary gland tissues, the supplements of retinoic acid and BMP4 are used to promote the aggregation of PSC, this is an essential step for the induction of PSC-derived organoids.^23^ Additionally, establishing PSC-derived organoids necessitates a controlled timing and sequence of adding exogenous molecules. Overall, it is important to stress the fact that the selection of best condition to grow oral and maxillofacial organoids needs to be set up case by case.

### Characterization strategies for organoids

Organoid systems should be characterized at morphological, gene expression, and functional levels, preferably with comparisons before and after cultivation to observe the effects of organoid development. Developing novel characterization strategies can better demonstrate the extent of mimicking the tissue and microenvironment of organoids, facilitating the establishment of a comprehensive standardized culture system and evaluation criteria. Traditional strategies for characterizing 3D organoids employ a small set of markers and heavily rely on histology, high-resolution microscopy, immunofluorescence, and bulk gene expression assays (e.g., bulk RNA sequencing and quantitative PCR).^60^

In morphological analysis, some current microscopy imaging techniques rely on molecular markers for analysis and cannot provide continuous observation and in situ tracking analysis of organoids. New imaging techniques and advanced data analysis methods have overcome these limitations and gain deeper insights into imaging data.^61,62^ Besides morphological observations, single-cell sequencing technologies can offer more precise comparisons of organoids with their source tissues at the level of cell types and gene expression patterns. Theoretically, in a single experiment, scRNA-seq enables impartial and thorough gene expression analysis of thousands of single cells. The technique is particularly useful for studying assembled organoid that contain multiple cell types. Various sequencing methods have recently been used to compare oral and maxillofacial organoids with traditional cell lines and tissues.^54,63,64^ Optimization of single-cell multi-omics techniques is expected to further promote the development of organoid characterization strategies compared to using only scRNA-seq. Simultaneous analysis of epigenomics, transcriptomics, proteomics, and other emerging omics such as spatial transcriptomics can better characterize organoids at the genomic scale.^62^ The availability of these technologies has improved our understanding of oral and maxillofacial organoids’ differentiation and has the potential to identify ways to further enhance organoid maturation. In terms of functional characterization, taking dental organoids as an example, the formation of calcium needs to be detected in order to identify enamel components.^24^ Salivary gland organoids are often transplanted into animals to test their salivary function.^65^

## Modified co-culture technology to generate assembled organoid

Supplement of scaffolds and/or molecules can only model the signal activation or inactivation within a single variable scenario, rather than providing the stem cells with their niche comprising stromal cells, vascular network and immune microenvironment. Recently, several bioengineering technologies have been employed to construct the organoid-stromal cells co-culture system, which is also called the assembled organoid.

### Cell clusters co-inoculation

This co-culture method can be divided into two sequential steps. Firstly, the generation of cell clusters, also called spheroids, is achieved through suspension culture. Among the simplest and most commonly used techniques is the low-adherence surface culture method, which fosters the formation of cell clusters capable of further development into organoids with tissue-like characteristics.^66^ Non-adherent culture conditions are conducive to the continued growth and differentiation of stem cells. Standard tissue culture dishes feature a negatively charged and hydrophilic surface; in contrast, the neutral hydrophilic gel-coated surface of ultra-low attachment plates significantly reduces the binding of adhesion proteins.^67^ Additionally, culture dishes treated with hydrophobic polymers, pluronic F-127, or bovine serum albumin can also provide low-adhesion surfaces to prevent cell adhesion.^68^ However, this method has some drawbacks, including difficulties in controlling the size of cell clusters and the inability to track the growth of each individual cluster. Other methods for cluster formation include hanging drop and spinner flask method, among others. Studies have reported that spheroids generated using the HD method exhibit denser structures compared to spheroids generated using another technique named molded parafilm-based.^69^ However, due to issues related to culture media and nutrient intake, HD spheroids display lower levels of differentiation. The spinner flask method, which has been used to generate human liver organoids, keeps cells in suspension by agitation, preventing cell adhesion to the substrate and promoting nutrient diffusion.^70^

The cell clusters or organoids formed by different stem cells through the above methods can be co-inoculated into the same organoid culture system and supplemented with growth-related signaling molecules to obtain the required assembled organoids. Some studies indicate that cell clusters co-inoculation techniques have provided a stable platform for direct cell-cell interactions.^5^ Recently, Harter et al. utilized solid 3D hydrogels to co-encapsulate intestinal organoids and peripheral blood mononuclear cells (PBMCs), demonstrating its capability to mimic crucial immune processes effectively.^71^ This co-culture technique has been applied to the construction of oral and maxillofacial assembled organoids. For instance, Yang et al. established a vascularized fibroblast-attached organoid (vFAO) model by using co-inoculation techniques to simulate the pathogenesis of oral submucous fibrosis (OSF), a malignant precancerous lesion in the oral mucosa.^72^

### Air–liquid interface (ALI) technology

The ALI technique, applied in 3D cultivation, entails culturing cells on a thin layer of microporous membrane, with the culture medium in contact only with the basal side of the membrane. This method has emerged as an effective approach for generating various epithelial organoids, such as kidney organoid,^73^ brain organoid,^74^ gastrointestinal organoid,^75^ lung and airway organoid.^76,77^ In comparison to the cell clusters co-inoculation, ALI technology affords enhanced oxygenation, thereby fostering organoid growth. Studies have demonstrated that cells cultured using ALI exhibit augmented cell–cell interactions and cell-stimulant interactions.^78^ Consequently, the utilization of this technique in co-culturing organoids is deemed an inevitable trend. Previously, Neal et al. employed an improved ALI technique to co-culture primary tumor epithelia with immune cells, including NK cells, T cells, B cells, and macrophages.^79^ This co-culture successfully recapitulated the tumor immune microenvironment and could be sustained for several months. Similarly, in modeling infection and inflammatory diseases, researchers have employed this technique to elucidate the interactions between human airway epithelial cells (HAE) and pulmonary microvascular endothelial cells (EC).^80^ Dobzanski et al. incorporated human sinonasal epithelial cells and fibroblasts into a co-culture system, constructing an assembled organoid model mimicking chronic rhinosinusitis with nasal polyps.^81^ By co-culturing patient-derived pancreatic ductal adenocarcinoma (PDAC) cells with mesenchymal cells and ECs derived from hiPSCs, Takeuchi et al. successfully generated assembled pancreatic cancer organoids at the air-liquid interface.^82^ In summary, co-culture systems based on ALI technology have successfully constructed assembled organoids comprising epithelial cells and other stromal cells, including fibroblasts, ECs, and immune cells.

### Microwell array technology

The microwell array technology can generate circular microwells of different diameters, controlling cell aggregation and the size and morphology of organoid. Currently, micropatterning techniques have been utilized for forming various organoids, including pancreatic organoids,^83^ intestinal organoids,^84^ renal organoids,^85^ hepatic organoids,^86^ with SG organoids being more commonly seen in oral organ engineering. However, this technique is rarely used in the construction of oral and maxillary assembled organoids. Kakni et al. developed a microwell-based intestinal organoid-macrophage co-culture system, which showed that macrophages attached to the inner surface of the microwell and maintained their normal cell phenotype. This novel intestinal co-culture organoid model provides insights into understanding mechanisms related to intestinal homeostasis and disorders, and also presents another technical possibility for the construction of oral and maxillary assembled organoids.

### 3D bioprinting technology

3D bioprinting, with its precision in controlling composition, spatial distribution, and structure of resulting organoids, may become a commonly used novel approach in the intersection of bioengineering and regenerative medicine.^87^ By depositing bioinks containing specific densities and types of cells, ECM, and other constituents, 3D bioprinting technology can generate organoids of different sizes, structures, and components with precise control. A nozzle-free acoustic droplet printing technique has been employed to construct oral cancer assembled organoids composed of cancer-associated fibroblasts (CAFs) surrounding tumor spheroids.^88^ Magnetic 3D bioprinting technology labels cells with magnetic nanoparticles and arranges them in space using magnetic dot arrays to rapidly produce 3D spheroids, thereby generating secretory epithelial organoids with neural innervation, such as salivary gland-like organoids derived from human dental pulp stem cells (hDPSCs).^65^

### Microfluidic devices

Microfluidic devices are significantly important for studying cell-cell interactions and facilitating the vascularization of organoids.^89^ The newly developed engineering co-culture system is also known as organs-on-chips.^90^ Platforms based on microfluidic chips provide open cavities in the form of one or more “microfluidic channels” for the fluid flow of cells, with the channel surfaces coated with ECM molecules to facilitate cell adhesion and further organoid formation.^91^ Moreover, the multicompartment design provides more controllable environments for co-culture of different cell types. Previously, a vascularized liver organoid was constructed using a microfluidic cell co-culture device.^92^ I this on-chip device, induced hepatic cells are co-cultured with ECs in a 3D ECM hydrogel. In addition to the construction of vascularized organoids, microfluidic devices have also made new progress in the co-culture of immune components. A microphysiological analysis platform for human brain organoids was developed to study immune-mediated brain aging.^93^ In this platform, primary monocytes were co-cultured with human cortical organoids to simulate neuro-immune interactions.

In summary, by combining the theoretical foundations of organ development, advanced ECM materials, and emerging organoid bioengineering technologies, the creation of more practical and precise assembled organoid models is within reach.

## Case-by-case strategies to generate assembled organoid using oral and maxillofacial tissues

Oral and maxillofacial region is particular in that it comprised not only soft tissue such as togue, salivary gland, and mucosa, but also hard tissue including tooth and bone. Generation of assembled organoid from this system would be much more complexed and may rely on an integrated application of biological and engineered approaches. Here, we retrospect the existing literature that described the construction of assembled organoids from oral and maxillofacial tissues, including tooth and soft tissues (Table 1). Furthermore, we evaluate the possibility to generate jawbone organoid by analyzing the current knowledge regarding to construction of bone organoid.

**Table 1:** Summary of exsisting construction strategies of tooth, mucosa and oral cancer assembled organoids based on co-culture technology (in chronological order)

Organoid type		Cell type	Ratio of cells	Scaffold	Characterization strategies	Reference
Tooth organoid	Tooth Root Organoid	DPSCs and PDLSCs	1:1	Scaffold-free engineered construct	Histological and microcomputed tomography analyses; quantitative image analysis of immunofluorescent staining, etc.	Calabrese TC et al.^18^
	Prevascularized dental pulp organoid	hDPSCs and Ecs	2:8	Matrigel	Hematoxylin-eosin staining and transmission electron microscopy imaging; using fluorescence microscope, etc.	Liu F et al.^100^
	IeAM organoid	ieAM cells derived from hiPSCs and hDPSCs	Not mentioned	Matrigel	Confocal imaging, etc.	Alghadeer A et al.^63^
	Human ameloblast organoid	hiPSCs and mouse dental mesenchyme	Not mentioned	Matrigel	Calcium imaging (Fluo-4 AM), confocal imaging, etc.	Kim KH et al.^24^
	Tooth assembloid	DESCs and DPSCs	Not mentioned	Matrigel	Single-cell transcriptomics, histochemical, immunostaining and TEM analysis, etc.	Hermans F et al.^44^
	Tooth germ organoid	hDPSCs and pDE	Not mentioned	GelMA HMPs	Using fluorescence microscope, etc.	Kilic Bektas C et al.^103^
	Tooth mesenchymal-epithelial organoid	ERM and DPSCs	2:1	Matrigel	Using transmission electron microscopy, scRNA-seq analysis, etc.	Hemeryck L et al.^43^
	Dental pulp organoid	hDPCs and ECs	3:1	hDP-ECM	Using a laser confocal microscope or fluorescence microscope, etc.	Xu X et al.^99^
Oral cancer organoid	OMM organoid	OMM cells and CD8^+^ TILs / CD45^+^ PBMCs	1:5 or 1:10	Matrigel	Histology and immunofluorescent staining, RNA-seq, organoid xenograft, etc.	Sun L et al. ^64^
	FAO	OSCC cells and CAFs	1:1	Matrigel	Immunofluorescence staining, etc.	Chen X et al.^22^
	OSCC organoid	OSCC cells and CAFs	1:1	Matrigel	Immunofluorescence staining, etc.	Zhao H et al. ^14^
	TSCC organoid	TSCC cells and CAFs	2:1	Matrigel	Using an inverted microscope, etc.	Almahmoudi R et al.^52^
OSF organoid	Vascularized FAO	oral epithelial cells, fibroblasts and HUVECs	1:3:1	Matrigel	Immunofluorescence, etc.	Yang X et al.^72^
SG organoid		SGOs and ADSCs	Not mentioned	BME	Proteomics analysis, etc.	Soto-Gamez A et al.^129^
		SGECs and mvECs	3:8	Decellularized porcine gut matrix (SIS-muc)	Immunofluorescence imaging, etc.	Burghartz M et al.^128^

*PDLSCs* periodontal ligament (PDL) stem/progenitor cells, *ieAM cells* induced early Ameloblast, *HERS cells* Hertwig’s epithelial root sheath cells, *GelMA* gelatin methacrylate, *pDECs* porcine dental epithelial cells, *hDPCs* human dental pulp cells, *HUVECs* human umbilical vein endothelial cells, *hiPSCs* human-induced pluripotent stem cells, *hAO* human ameloblast organoid, *ECs* endothelial cells, *hDP-ECM* human dental pulp derived extracellular matrix, *OMM* oral mucosal melanoma, *TILs* tumor-infiltrating lymphocytes, *PBMCs* peripheral blood mononuclear cells, *FAO* fibroblast-attached organoid, *OSCC* oral squamous cell carcinoma, *CAFs* cancer-associated fibroblasts, *CSCs* cancer stem cells, *TSCC* tongue squamous cell carcinoma, *OSF* oral submucous fibrosis, *ADSCs* adipose-derived mesenchymal stem cells, *BME* basement membrane extract, *SGOs* salivary gland organoids, *SGECs* salivary gland epithelial cells, *mvECs* microvascular endothelial cells

### Tooth organoid

Teeth, the hardest structures within the human body, represent highly intricate mineralized organs. Particularly, natural teeth consist of four distinct components with varying degrees of hardness: enamel, dentin, cementum, and dental pulp. This implies that faithfully replicating a natural tooth is a formidable challenge. Tooth organoids are expected to develop tooth-like structures with comparable hardness and elastic modulus to natural teeth, containing dental pulp tissue to provide space for vascularization and neural regeneration. In the realm of teeth and periodontal tissues, diseases such as pulpitis, apical periodontitis, periodontitis, and the eventual tooth loss have a profound impact on both oral and systemic health.^1^ Tooth organoids (including dental pulp organoids, dentin-pulp organoids, dental germ organoids, etc.) have emerged as progressively pivotal models mimicking the progression of the aforementioned disease.^94,95^

Previous studies have reported that 3D dental pulp organoids can be used to study pulp diseases or regenerate pulp treatments.^96^ Dental pulp, composed of fibroblasts, mesenchymal cells, dentin forming odontoblast cells and immune cells, is a highly vascularized and innervated connective tissue.^97^ Due to the complexity of dental pulp tissue, it is inevitable to reconstruct a microenvironment similar to natural dental pulp in order to induce odontogenic mesenchymal stem cells (MSCs) to generate assembled organoids containing dental-pulp-like structures.^98^ A novel 3D dental pulp organoid model was developed using human dental pulp-derived ECM to simulate the specific microenvironment of endodontic tissue. Functional pulp organoids were formed by co-culture of human dental pulp cells (hDPCs) and ECs.^99^ Prevascularized dental pulp organoids were established to elucidate mechanisms of angiogenesis in the process of pulp formation.^100^ In future studies, a certain amount of nerve cells and immune cells should be added to optimize the dental pulp organoid model.

Tooth organoids are primarily formed by the time- and space-dependent interactions between the epithelium and mesenchyme, orchestrated by relevant signal molecules. Growing numbers of studies in recent years have concentrated on the formation of tooth germ organoids. The earliest tooth germ organoid originated from DE and DM cells isolated from mouse mandibular embryonic tooth germ and co-cultured in collagen gel, which were transplanted into alveolar fossa to produce bioengineered teeth.^101^ Subsequently, similar approaches successfully yielded tooth germ organoids in canine and porcine models.^28,102^ Hemeryck et al. established epithelial organoids derived from human dental follicles tissue, sourced from unerupted wisdom teeth.^43^ These were combined with dental mesenchymal cells (e.g., DPSCs) to form assembled organoids, inducing ameloblast differentiation and enamel formation. Furthermore, hAOs derived from hiPSCs exhibited similar properties to ameloblasts, secreting enamel and demonstrating the capacity to mineralize teeth through interactions with dental mesenchyme.^24^ Recently, tooth root organoids were constructed by co-culturing DPSCs and PDLSCs.^18^ Other tooth assembled organoids are listed in Table 1, illustrating their construction conditions and characterization strategies.^63,103^

In conclusion, these advancements in strategy optimization exemplify the transition from dental pulp organoids and tooth germ organoids to well-structured bioengineered teeth. Currently, various strategies for in vitro culture of tooth organoids do not yet fully replicate the entire tooth structure, including hard tissues (enamel, dentin, cementum), dental pulp, vasculature, and nerves. Further development and assembly are achieved post-transplantation in animal models. It is necessary to explore more effective culture methods to promote the development of disease simulation and dental regeneration.

### Jawbone organoid

It is widely acknowledged that jaw bone, comprised of maxilla and mandible, incorporate mineralized osseous tissue and bone marrow, along with traversing vasculature and nerves. Bone defects are often caused by bone fracture, jaw osteomyelitis, jawbone tumor and other diseases, and difficult to be completely cured due to the inherent limitations of treatment modalities. Currently, the main therapeutic approaches for bone augmentation often relies upon autogenous or allogeneic bone graft transplantation. Nonetheless, the availability of these tissues is constrained, and potential adverse biological reactions curtail the scope of clinical utility. Many kinds of skeletal system organoids, including woven bone, bone marrow, osteochondral, cartilaginous, and callus organoids, have been created by researchers over the years, ranging in complexity.^104^ These innovations hold promise for application in the treatment of oral osseous tissue disorders. In principle, the construction of skeletal system organoid in vitro emulates the skeletal development events in vivo, culminating in the attainment of bone regeneration. Regenerated bone tissue has bone-specific functions and is expected to potentially supplant conventional bone grafts.

Existing skeletal system organoids are limited to displaying a single function of bone, including bone formation, resorption, or hematopoiesis, thereby inadequately recapitulating the dynamic processes in the bone niche. Studies on skeletal organoids are rarely applied in the treatment of oral bone tissue diseases. However, given the analogies in bone development and the predominant use of autogenous bone grafts in the restoration of osseous defects (e.g., autologous iliac bone grafting),^105^ it is suggested that skeletal organoids and bone regenerative medicine possess a wide range of potential application in the treatment of oral-maxillofacial diseases. The cultivation and development of maxillary and mandibular organoids hold potential to offer enhanced curative treatment for patients with jaw bone defects, such as those resulting from post-cancer resections.

Comparable to soft-tissue organoids, the culture of skeletal organoids is also intrinsically reliant upon three elements: stem cells, scaffolds, and endogenous-exogenous signals. Many skeletal organoids are sourced from murine osteogenic precursor cells (e.g., bone marrow stem cells [BMSCs] and skeletal stem cells), human periosteum-derived cells,^106^ and the frequently deployed MSCs for composite organogenesis. In terms of scaffolds, considering the significant difference in hardness between bone tissue and soft tissue, the behaviors and functions of bone cells will be affected by the mechanical properties of the matrix microenvironment, and more attention should be paid to the mechanical and biochemical characteristics of the culture materials. Matrigel’s mechanical properties are poor and non-adjustable, limiting its development in skeletal organoids. Collagen, gelatin, and demineralized bone matrices have been explored for skeletal organoids. Discrepancies in endogenous and exogenous signals are represented by bone morphogenetic protein 2 (BMP2, expediting osteogenic differentiation) and receptor activator for nuclear factor-κB ligand (RANKL, promoting osteoclast differentiation). Furthermore, sustained mechanical loading has demonstrated a positive influence on skeletal organogenesis.^107^

To date, a variety of skeletal organoids have been successfully developed, and skeletal organoids have also been developed in a more complex direction. In fact, bone tissue has a unique multifunctional unit structure (bone forming unit, bone resorption unit, and hematopoietic unit), and does not have epithelial components. Assembled skeletal organoids frequently tend to have multiple type of cells, such as co-culture of osteoblasts and osteoclasts. Park et al. utilized decellularized bone paper to provide a biochemical environment comparable to the bone ECM.^108^ Osteoblasts were induced to deposit structural mineralization of bone tissue and to obtain the phenotype of bone lining cells. Subsequent co-culture of the established osteoblastic lining cells with bone marrow mononuclear cells effectively induced their differentiation into osteoclasts. This in vitro trabecular bone organoid reproduces the bone remodeling cycle. Additionally, woven bone organoid has been established through co-culture of hBMSCs differentiated into osteoblasts and osteocytes.^109^ Analogous to assembled soft-tissue organoids, the incorporation of ECs in skeletal organoids has been explored to simulate vascularized tissue-like structures. Blache et al. co-cultured MSCs with ECs in round-bottomed PEG hydrogel microwells to generate bone marrow organoids with functional characteristics in vivo.^110^ Recently, successful production of a neural-bone construct through multicellular 3D bioprinting technology has been reported, signifying their potential as innervated-bone organoids. The upper neurogenic cells (Schwann cells) distributed in the construct and the bottom osteoblasts (BMSCs) were co-cultured in the bioinks, which are matrix of GelMA integrated with calcium silicate nanowires. Finally, organoids were implanted into rats with skull defects, showing successful regeneration of innervated bone.^111^ Current research in jawbone regeneration is predominantly confined to animal models, with the imminent prospect of using iPSCs and BMSCs to construct human-derived jawbone organoids. With the help of the advanced techniques, such as 3D printing and microfluidics, it is possible to construct vascularized and immune cell-integrated organoids and promote the development of jaw defect repair and treatment.

Temporomandibular joint (TMJ), recognized as one of the most intricate joints in the human body, plays an important role in essential activities such as chewing, speaking, and breathing. However, research in the field of TMJ-related organoid studies remains relatively sparse. Unlike the knee joint, the development of TMJ prostheses has been slow and contentious. Given the proximity of the joint to crucial sensory nerves, the inner ear, and the brain, implant failure could result in catastrophic consequences, including particle migration, nerve damage, and cranial fracture. Organoids of bone and cartilage associated with the TMJ offer novel possibilities for studying and treating temporomandibular joint diseases, propelling advancements in the TMJ field. Abraham et al. combined cell derivatives isolated from bone and cartilage tissues with ECs to construct bone and cartilage organoids.^112^ Then they built bone-cartilage organoids by co-culture the cell derivatives harvested from rib tissue containing bone and cartilage, which they called “mini-joint” models. The maturation of 3D printing systems for miniature knee joints may also guide the development of TMJ organoids in disease progression and drug development. This system, also known as “mini-joint”, comprises osteochondral complex, adipose tissue, and synovial-like fibrous tissue assembled into miniature joint chambers.^113^ Human bone marrow-derived mesenchymal stem cells (hBM-MSCs) serve as the cell source for this system. Additionally, research indicates that iPSCs can be induced into mesenchymal progenitor cells (iMPCs), generating bone-cartilage tissue chips by optimizing iMPCs’ cartilage and bone induction conditions, applicable for osteoarthritis modeling.^114^

Presently, the evolution of skeletal organoids remains in its nascency, with the majority of organoids eventually evaluated through in vivo implantation to test their regenerative potential for osseous tissue. Therefore, some researchers put forward the concept of in vivo osteo-organoid, which simulated several different stages of development.^115^ A recent case report has confirmed the clinical translational capacity of in vivo bone organoid strategies in reconstructing large maxillary defects.^116^ However, the application of in vivo organoids in regenerative medicine and translational medicine needs further research due to the many ethical issues involved in the construction of animal models, the long culture cycle and the possible immunogenicity of the obtained organoids.

### Oral cancer and mucosal organoid

OSCC is a common malignant tumor occurring in the oral mucosa, lip and tongue, representing 90% of all forms of oral cancer.^117^ It is one of the leading causes of incidence and mortality worldwide.^4^ Oral carcinogenesis is a multistep process, and despite significant advances in existing research, our understanding of the disease remains incomplete. The emergence of oral cancer organoids (including tongue cancer organoids) brings novel possibilities for basic tumor research, disease simulation, drug development and precision therapy. At the same time, oral cancer assembled organoids need to be developed to emulate the tumor microenvironment (TME) more accurately, allowing the preservation of genetic, molecular, structural, and functional characteristics of tumor cells in the highly dynamic and complex TME.

Oral mucosal organoids can serve as possible platforms for personalized cancer treatment and may mimic the pathophysiology of normal mucosa after viral infection. Research by Driehuis et al. confirmed the effective infection of oral mucosal organoids by HSV1 and HPV16, enabling the simulation of virus-related diseases such as oral herpes.^53^ HPV, recognized as a risk factor for oral cancer, contributes to tumorigenesis in the OSCC tumor subgroup.^118^ The infection of oral mucosal organoids with HPV offers a promising avenue to investigate tumor cell genetic alterations and interaction between cells and the microenvironment during carcinogenic transformation. Heller et al. co-cultured human gingival epithelial cells, fibroblasts and microvascular endothelial cells (mvECs) to generate a pre-vascularized buccal mucosa equivalent.^119^ The capillary-like structure was formed and anastomosed with the host vasculature to become functional vessels after implantation in nude mice. Although this model has not been applied to oral cancer research, it seems to provide new ideas for the construction of assembled organoids for oral cancer. OSCC usually originates from precancerous lesions of the oral mucosa, such as oral submucous fibrosis, oral leukoplakia and oral erythroplakia. However, due to limited research on precancerous lesions and a concentration of models in 2D cell culture, future applications of organoid models may clarify the mechanisms of malignant transformation in precancerous lesions.

**Fig. 3:**
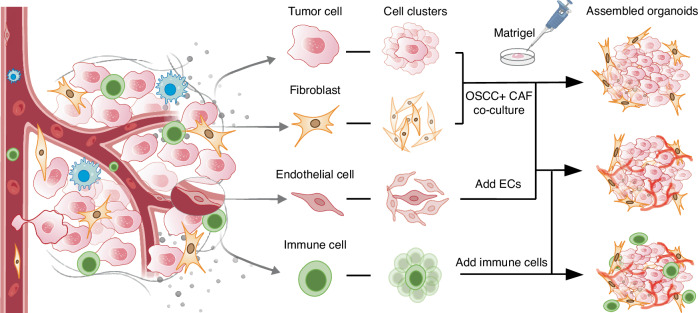
Cell clusters co-inoculation for culturing assembled oral cancer organoids. Different types of cells derived from the tumor microenvironment are cultured into cell clusters, which were added into the scaffold containing growth factors to construct assembled organoids. Future study may shed more light on the construction of the vascularized organoids and the infiltration of immune cells into oral cancer organoids

Given the important role of cancer-associated fibroblasts (CAFs) in promoting tumor-stromal crosstalk, the construction of CAF-related tumor assembled organoids is of great significance for the development of oral cancer organoids. By co-culturing CAF with oral cancer organoids, Zhao et al. increased the longevity of cancer organoids and investigated the function of fibroblasts in tumorigenesis.^120^ Findings showed that the 3D co-culture model promotes the stem-like properties of primary OSCC cells, improving the organoids-forming ability and promoting tumor progression by CAF-derived lactate. Then the fibroblast-attached organoid model was used to study the phenotypic transition of fibroblasts during tumor-stromal interactions.^22^ The important role of CAFs is equally emphasized in advancing the basic research of tongue squamous cell carcinoma (TSCC) and the construction of related organoids. Zhao et al. co-cultured TSCC cell line Cal27 and CAFs using decellularized tongue extracellular matrix.^121^ Another CAF-associated TSCC organoid demonstrated that the presence of CAF increases cancer cell aggressiveness.^52^ Future investigations may unveil an efficient trinity of assembled organoids composed of OSCC cells, CAFs, and ECs, offering new possibilities for the vascularization of tumor organoids (Fig. 3). Simultaneously, the differentiation of iPSC into abundant ECs could enhance the cellular source for tumor assembled organoids. Engineering approaches such as 3D printing and microfluidic technologies could be employed to construct vascularized tumor organoids, further optimizing the construction of oral cancer assembled organoids. Infiltration of immune cells into oral cancer organoids may be the next research direction.^64^ Moreover, establishing patient-derived organoid-based xenografts (PDOX) offers a promising avenue by integrating in vitro organoid studies with animal models, significantly advancing the field of precision medicine. Although the application of PDOX models in oral cancer research is currently limited, their unique advantages are undeniable. These include high fidelity to the original tumor, providing more comprehensive in vivo and in vitro paired data, increased drug sensitivity accuracy, and higher transplantation success rates. A series of PDOX models, including colorectal cancer,^122^ liver cancer,^123^ ovarian cancer^124^ and so on, have been successfully established. PDOX, serving as clinical trial models, plays a crucial role in screening anti-tumor drugs, identifying biomarkers, and personalized therapies.

### Other soft-tissue organoids

Apart from mucosa, the soft tissue organs of the oral and maxillofacial region mainly include salivary glands and tongue, but so far, the research on assembled organoids in these two aspects is relatively rare. The salivary glands are made up of acini and ducts. The acinar is the main secretory unit of SG parenchyma, formed by serous and mucous acinar cells alone or in combination, which produce and secrete saliva. The SG microenvironment includes the surrounding basement membrane and ECM, various immune cell populations, adipose and muscle tissue, the autonomic nervous system and a complex vasculature.^125^ Some mesenchymal cells are used to induce the formation of salivary gland epithelial organoids. For instance, BM-MSCs induced self-assembly and branching of primary epithelial cells in 3D culture.^126^ Ultimately, an EC/MSC combined organoid fabricated in a biomimetic environment and had unique cellular structure. In addition, some studies have shown that DPSC may promote the differentiation of acini, providing a new possibility for SG tissue regeneration.^127^ In vascularization of salivary gland organoids, mvECs can be co-cultured with salivary gland cells but are not sufficient to form functional networks.^128^ Soto-Gamez et al. co-cultured adipose-derived MSCs and SG organoids to promote the ex vivo expansion of SG stem cells.^129^ It is well-known that hyposalivation arises from factors such as inflammation, aging, medication side effects, and off-target radiation in the treatment of head and neck tumors. Hyposalivation can further lead to oral-maxillofacial disorders and even systemic diseases. Presently, organoid models have been developed to simulate conditions like xerostomia,^65^ SG inflammation,^130^ and radiation-induced damage,^131^ proving to be effective in elucidating the mechanisms underlying SG dysfunctions in vitro.

At present, more research focuses on the construction of taste bud organoids and tongue epithelial organoids, rather than assembled organoids. Four types of papillae—filiform, foliate, fungiform and circumvallate papillae—are found in the lingual dorsal epithelium.^15^ And foliate, fungiform and circumvallate papillae have gustatory buds, whereas filiform papillae do not. However, a study has integrated taste bud organoids transplantation into the lingual epithelium of the recipient mice, and found that suspension-cultured taste bud organoids have the potential for implantation and innervation after tongue transplantation.^132^ Moreover, single-cell RNA sequencing (scRNA-seq) revealed that this organoid contained lingual epithelial cells, taste receptor cells, taste precursor cells and taste stem cells. Furthermore, taste bud organoids have been employed to construct disease models for radiation-induced oral mucositis, validated through murine experiments.^133^

## Limitations and challenges of construct oral and maxillofacial organoids

The incorporation of environmental components into assembled organoid system, including vascular, neural and immune population, has become a central issue in the field of oral and maxillofacial researches. There are many limitations and challenges to be further addressed in this process.

### Vascularization of oral and maxillofacial organoids

**Fig. 4:**
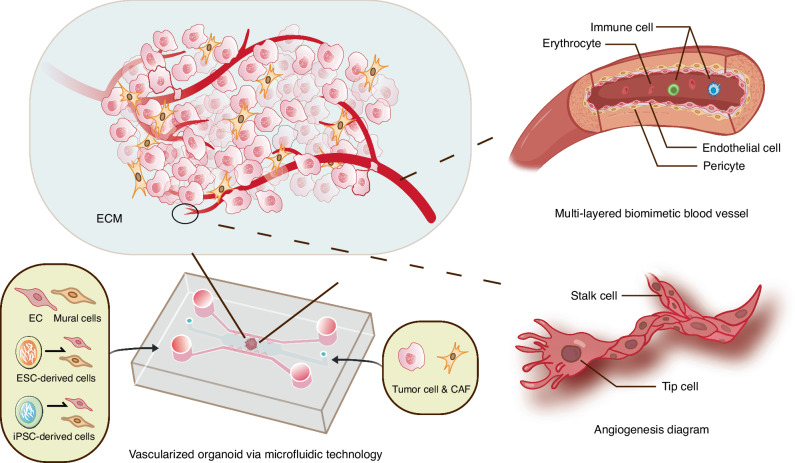
Vascularized oral cancer organoids constructed by engineering technologies (take microfluidics for example). The dotted lines represent that relevant research still needs to be further developed, including the construction of multi-layered biomimetic blood vessels and the dynamic process of vascular sprouting

It is widely known that the tissues within oral and maxillofacial region are densely infiltrated with blood vessel networks, enabling the provision of nourishment and eradication of metabolic waste that underlie both homeostasis and pathogenesis of oral tissues. The absence of vasculature in typical organoid system may give rise to their growth limitation during in vitro expansion, thereby challenging the comprehensive disease modeling for parental tissues by organoid technologies. The common approach to vascularizing organoids involves implantation into highly vascularized animal tissues. However, the success rate of such transplants is modest, necessitating co-cultivation of quasi-organs with stromal and ECs to mitigate implantation failures.^134^ To solve this problem, researchers have begun to try to integrate vascular endothelial cells into the microenvironment of organoids to promote the formation of vascular structures. Engineering technologies such as micromolding, microfluidics, 3D printing and electrospinning can be used to artificially construct vessel‐like structures in vitro tissues to achieve the vascularization of organoids (Fig. 4).

Although the existing organoid vascularization method has been well proved and has achieved initial results in the field of application, the road to practical application is still very long.^135,136^ For the vascularization of different oral and maxillofacial organoids, the specificity of ECs should be considered, avoiding over-simplified use of HUVECs. Except for HUVECs, other source of ECs, such as the EPCs and hPSC-derived ECs (hPSC-ECs), have also been employed to model the angiogenesis in vitro.^137^ EPCs mostly exist in embryonic tissues, showing strong angiogenesis potential, high proliferation capacity and high plasticity.^138^ However, the insufficient source of EPCs has become an urgent problem to be solved. In contrast, hPSC-ECs from the same donor can provide an approximately inexhaustible supply of ECs and be used for personalized and precision treatment.^139^ In addition, the development of vascular system obviously involves more than ECs, which constitute the inner wall of blood vessels. To construct functional biomimetic blood vessels, it is necessary to refine and optimize the in vitro construction strategy. The inclusion of other cell types, such as mural cells (pericytes in smaller vessels and vascular smooth muscle cells in larger-diameter vessels), provides structural and functional support for organoid angiogenesis. These cells can also be derived from hPSCs.^140,141^ Focusing on the role and behavior of tip cells and stalk cells (subtypes of ECs)^142^ in the dynamic process of vascular sprouting also provides an important direction for the study of vascularized organoids.^143^ At the same time, the stability of microvessels can be improved by gene editing technology.^144^ Presently, the research on vascularized organoids focuses on organs with rich blood supply, that is, the organs with more active metabolism, such as brain,^142^ heart,^145^ bone marrow^146^ and so on.^147^ The research on oral and maxillofacial organoids needs to be further deepened. In addition, the vascularization of organoids has great influence on the establishment of oral cancer organoid model. It provides nutrients for tumor growth, promotes the metastatic spread of cancer cells, and also affects the transport and function of immune cells. Vascularized tumor organoids can better simulate the complex TME, which is conducive to disease research and treatment. More attention should be paid to the application of vascularized organoids in the construction of oral cancer models.

It is still a great challenge to construct multi-layered biomimetic blood vessels and build more controllable and predictable self-assembly blood vessel networks in vitro, providing a more comprehensive platform for the development of vascularized organoids. Advanced biomaterials and novel dynamic systems should be developed in the long run. Eventually, vascularized organoids with mature biological functions will be widely used in regenerative medicine and personalized therapy.

### Integration of immune cells into oral and maxillofacial organoids

During the pathophysiology of a vast majority of oral diseases, particularly the periodontitis and OSCC, the immune cells are commonly recruited into disease-related microenvironment, and play dual role in promotion and suppression of their progress. The disease modeling and drug screening for immune-related disease and immunotherapy, to date, remain extremely tricky at the in vitro level, due to the lack of preclinical model with intact immune microenvironment, and are drastically dependent on in vivo model, which may cost several years for stable manipulation. In the field of oncology research, specifically, the advance in organoids technologies did provide a brand-new strategy to model the cancer-associated immune system for individual patients. In the PDO model, the immuno-tumor microenvironment can be constructed in several ways^148^: (1) In assembled organoids, tumor cells derived from the patient’s tumor tissue are co-cultured with immune cells derived from peripheral sources (i.e., peripheral blood, lymph nodes) or tumors (i.e., tumor-infiltrating lymphocytes (TILs)); (2) Several PDOs retain natural tumor-infiltrating immune cells upon isolation; (3) Transplanting PDOs into animal models induces a process of immune system reconstitution, wherein immune cells infiltrate and populate the PDO.^149^ For instance, the organotypic tumor spheroid MDOTS/PDOTS isolated by Jenkins et al. from mouse and human tumors retained autologous immune cells and lymphocytes.^150^ The spheroid-collagen mixture was then injected into a 3D microfluidic device to culture the assembled tumor organoid. Another culture platform is air–liquid interface (ALI). Neal et al. described an ALI tumor organoid for human in vitro immunotherapy modeling by unified culture of tumor epithelium and its homologous TIL.^79^ These methods have great potential for modeling and understanding immune responses in the tumor microenvironment.

For the integration of immune cells and oral organoids, there are still some problems to be solved.^151^ First, the dense structure of the hydrogel may affect the infiltration of immune cells. Second, because organoids and immune cells have different needs for different stem cell signaling factors, there is currently no universal expansion medium for neither immune cells nor organoids. Precise experiments should be performed before attempting co-culture to discover the optimal medium in which immune cells are not harmed and organoids are still able to proliferate. In addition, the growth of assembled organoids with immune cells may be limited by the influence of immune cells. Simulation of the immune environment is another challenge in organoid research, especially in simulating the interaction between organoids and the immune environment. However, PDOs that combine immunity and other stromal components could help realize the promise of precision medicine and personalized treatments.

### Gene editing

Gene editing technologies applied in the organoid field have some potential risks and limitations. For instance, off-target effects, immunogenicity and cellular toxicity in response to the DNA damage are still several challenges that limit the development of gene editing.^152^ Compared to 2D culture, the complex conditions required for organoid culture pose challenges in delivering genome editing agents to organoids. In HEK293T cells, transfection efficiency with plasmid DNA higher than in 3D organoidsg.^153^ In the future, improving the safety and efficiency of CRISPR editing in human stem cells and optimizing the delivery of editing agents to organoids are crucial directions for future research. Advanced genome editing tools, such as non-DSB genome editing (base editing^154^ and prime editing^155^), offer the potential to minimize off-target effects and enhance editing precision. Innovative delivery strategies, such as nanoparticle-mediated delivery, may facilitate effective introduction of editing agents into organoids.^156^ By addressing these challenges, we can fully harness the potential of gene editing technologies to uncover the complexity of development and disease progression in OMR.

## Clinical applications of oral and maxillofacial organoids

**Fig. 5:**
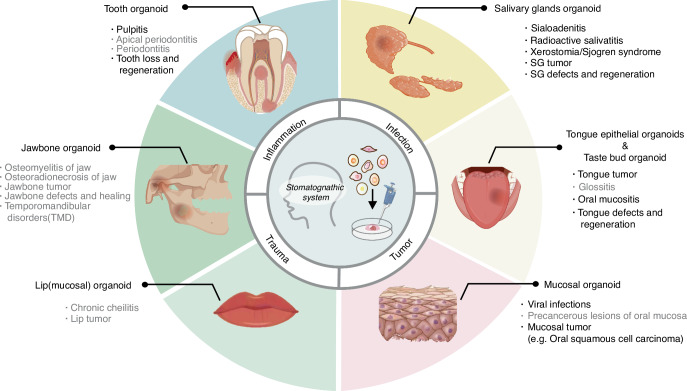
Application of oral and maxillofacial organoids in the simulation of related diseases. Associated diseases include the following four main types: inflammation, infections, trauma, and tumors. Organoids are categorized into six types depending on the different types of organs. Diseases with black letters have been mimicked by related organoids, but in gray font are relatively rarely reported

Oral and maxillofacial diseases are a wide spectrum of illness that may damage multiple organs including tooth, alveolar bone, mucosa and salivary gland. Since organoids can be generated in a tissue-specific manner, the organoid technologies may serve as a powerful strategy to benefit clinical treatment of this intricate entity. There, we summarize the application of the oral and maxillofacial assembled organoids mentioned above in the field of disease simulation (Fig. 5). From bench to bedside, we focus on the potential of these organoids in clinical applications, including regeneration, drug screening, among others.

### Tooth organoids

Unlike mice, when human teeth are fully formed, the ameloblast that form tooth enamel undergo apoptosis.^63,157^ Therefore, the human body can’t repair or regenerate the damaged tooth enamel, which affects the chewing function, resulting in tooth sensitivity, dental caries and even teeth loss. Presently, dental implants serve as commonplace alternatives to natural teeth. However, their implantation can be accompanied by a series of severe complications, such as peri-implantitis, gingival and periodontal tissue recession, bone resorption, and ultimate implant failure.^158,159^ It is hoped that advances in tooth regeneration engineering will lead to the generation of bioengineered teeth (including dental organoids), that will eventually be sufficient to replace conventional dental implants. Although tooth germ organoids used today have not yet grown into perfect teeth with mature structures in vitro, they can produce functional teeth after transplantation into the jaw.^160^ Researchers are directing their efforts towards inducing enamel formation in vitro using tooth organoid models, paving the way for future dental regenerative medicine. Hemeryck et al. established a mesenchyme-epithelium assembled organoid model, demonstrating that DPSCs trigger epithelial stem cells to differentiate into ameloblasts.^43^ The hAOs showed potential for tooth regeneration when interacting with mouse dental mesenchyme, forming mineralized tooth-like structures upon transplantation.^24^ The ieAM organoids, after both in vitro culture and in vivo transplantation, expressed essential enamel proteins required for mineralization.^63^ Vascularization of dental pulp organs also holds promise for dental pulp-dentin regeneration, yet significant progress is needed for clinical applications.^100^

### SG organoids

Salivary gland regeneration can improve salivary gland function, addressing saliva secretion disorders caused by aging, injury, Sjögren’s syndrome, and radiation therapy. Currently, researchers have successfully cultivated salivary gland organoids capable of saliva secretion using traditional cell culture techniques or advanced 3D bioprinting.^45,161^ These organoids, when transplanted into damaged salivary glands, significantly stimulate the regeneration and growth of damaged glandular tissue epithelium and neurons.^65^ Using SG organoids, researchers study stem cell radiation response mechanisms to simulate insufficient saliva secretion due to radiation damage, in order to better treat radiation-induced xerostomia in head and neck cancer radiotherapy patients.^162^ Yoon et al. develop a long-term organoid culture method maintaining salivary gland organ phenotypic characteristics, which provides an important tool and platform for further exploration of salivary gland regenerative medicine.^54^ Overall, salivary gland regeneration technology holds promise as an effective approach to treating salivary gland dysfunction, offering new options to improve patients’ quality of life.

### Mucosal and taste bud organoids

The construction of oral mucosal organoids holds promise in providing significant assistance in treating viral infections and potential malignant diseases, while offering new research avenues for the diagnosis and treatment targets of early-stage malignant diseases. Currently, clinical models for premalignant diseases such as oral leukoplakia, erythroplakia, and OSF are relatively scarce, which limits research and development of treatment methods for these diseases. Recently, the vFAO model has demonstrated new research strategies in studying the pathogenesis of OSF and has shown greater clinical relevance, although its specific application in clinical settings requires further exploration and validation.^72^ Salahudeen et al. used oral mucosal organoids to screen candidate oncogenic drivers. The study revealed that DYRK2 kinase at the 12q15 as an amplified HNSCC oncogene in p53^−/−^ oral mucosal organoids.^163^ These findings provide new insights into the molecular mechanisms of oral mucosal diseases, laying the foundation for future development of targeted therapeutic strategies and early diagnostic methods.

The loss of taste severely affects the quality of life of patients. Taste bud organoids offer hope for solving this puzzle. The researchers extracted taste bud organoids from a mouse model of oral mucositis to study the therapeutic effect of drugs on taste loss.^133^ At present, a variety of taste bud organoids have been successfully constructed. For example, taste bud organoids are cultured in suspension, integrated with the recipient tongue epithelium after transplantation for regenerative medicine, and microfluidic taste devices with taste cells and tongue-like microenvironments are used to construct artificial tongues.^132,164^ Recently, Wu et al. constructed a Taste Organoids-on-a-Chip System, hoping that this bioelectronic tongue can facilitate studies in disease modeling and drug screening.

### Oral cancer organoids

PDOs serve as an important bridge between animal models and human clinical trials due to their ability to faithfully summarize the physiopathology of different patients. They can be used as patient avatars to test drug responses, essentially becoming personalized drug testing models. Ultimately, they can assist clinicians in selecting more appropriate treatment options, optimizing efficacy, and reducing side effects in individual patients. There are numerous tumors occurring in the OMR, and organoid models tailored for these tumors have been developed and applied in drug screening and new drug development. Driehuis et al. devised protocols for establishing organoids from various epithelial tissues and cancers, including OSCC, providing platforms for subsequent drug screening.^165^ Organoid models derived from circulating tumor cells in the blood of HNSCC patients before treatment were used to detect their sensitivity to drugs such as cisplatin, docetaxel, and 5-fluorouracil, achieving a treatment prediction accuracy of 93.75% when combined with mathematical models.^166^ Additionally, Czerwonka et al. tested the efficacy of a new generation of small molecule Notch modulators represented by RIN-1 and CB-103 using HNSCC organoids, demonstrating that RIN-1 could serve as a chemotherapy agent for patients with intact Notch signaling.^167^ OMM organoids showed significant similarities and differences in relative sensitivity to receptor tyrosine kinase (RTK) multi-target inhibitors and chemotherapy drugs, including erlotinib and paclitaxel, proving their utility as preclinical models for analyzing drug responses in relation to genomic and transcriptomic changes. Multiple studies have validated the drug screening capabilities of salivary gland cancer (SGC) organoids.^168–170^ Recently, Ishikawa et al. confirmed that organoids can be used to validate oncogenic pathway activation and test genotype-guided molecular targeted therapies, potentially enhancing precision medicine efficacy for SGC patients.^170^ Overall, these studies demonstrate the diverse applications of organoid models in oral and maxillofacial tumor research, offering new prospects for optimizing personalized medical and treatment strategies in the future.
