# Author Correction: Organoids in the oral and maxillofacial region: present and future

**DOI:** 10.1038/s41368-025-00377-5

**Published:** 2025-05-27

**Authors:** Yufei Wu, Xiang Li, Hanzhe Liu, Xiao Yang, Rui Li, Hui Zhao, Zhengjun Shang

**Affiliations:** 1 State Key Laboratory of Oral & Maxillofacial Reconstruction and Regeneration, Key Laboratory of Oral Biomedicine Ministry of Education, Hubei Key Laboratory of Stomatology, School & Hospital of Stomatology, Wuhan University, Wuhan, China; 2 Department of Oral and Maxillofacial-Head and Neck Oncology, School of Stomatology–Hospital of Stomatology, Wuhan University, Wuhan, China

**Keywords:** Cancer models, Regeneration, Tissue engineering

Correction to: *International Journal of Oral Science* **16**: 61 (2024); 10.1038/s41368-024-00324-w, published online 01 November 2024

Following publication of the original article,^1^ the authors reported two issues in the article:

Issue 1: Reference misalignment in Fig. 2. During the proof revision stage, the removal of duplicate references inadvertently caused a misalignment of reference numbers in Fig. 2.

The originally published Fig. 2 was:
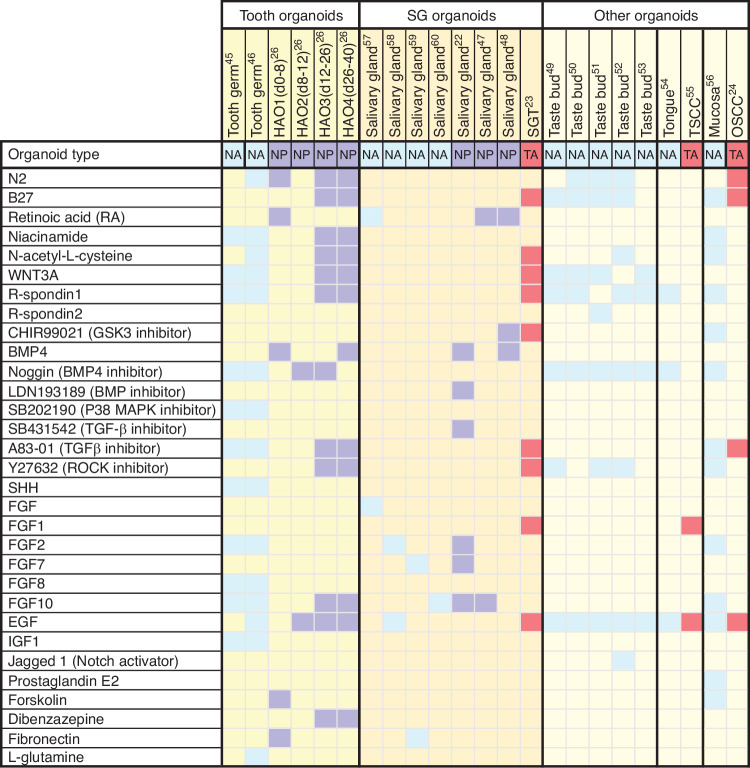


**Fig. 2** Heatmap showing the culture conditions for various oral and maxillofacial organoid cultures. Each column shows a culture condition protocol that has been reported for a particular type of organoid. Colored boxes signify the indicated growth factor component used in organoid expansion protocols. The organoid types were divided into NA, normal tissue ASC-derived organoids, NP, normal tissue PSC-derived organoids, and TA, tumor ASC-derived organoids. hAO1-4, different culture stages of the human ameloblast organoids; SGT, salivary gland tumor; TSCC, tongue squamous cell carcinoma; OSCC, oral squamous cell carcinoma

The correct Fig. 2 should be:
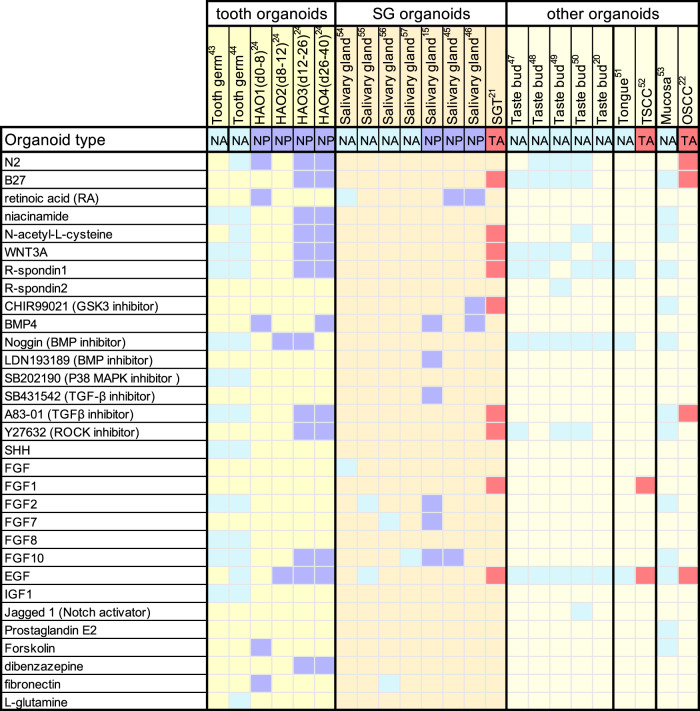


**Fig. 2** Heatmap showing the culture conditions for various oral and maxillofacial organoid cultures. Each column shows a culture condition protocol that has been reported for a particular type of organoid. Colored boxes signify the indicated growth factor component used in organoid expansion protocols. The organoid types were divided into NA, normal tissue ASC-derived organoids, NP, normal tissue PSC-derived organoids, and TA, tumor ASC-derived organoids. hAO1-4, different culture stages of the human ameloblast organoids; SGT, salivary gland tumor; TSCC, tongue squamous cell carcinoma; OSCC, oral squamous cell carcinoma

Issue 2: Text Revision in Section CONSTRUCTION AND CHARACTERIZATION OF ORAL AND MAXILLOFACIAL ORGANOIDS. The authors propose a revision to clarify a statement in the “Supplement molecules” paragraph under “Three key elements to construct oral and maxillofacial organoids”. This adjustment emphasizes the specificity of molecular requirements across organoid types while retaining the original reference identifiers unchanged.

The original text was:

For instance, WNT signal-related proteins, such as WNT3A and Rspondins, are essential for tooth and mucosa organoids but not for salivary gland organoids.

The revised text should be:

For instance, the WNT pathway-related proteins, such as WNT3A or Rspondins, are essential for tooth and mucosa organoids but not for salivary gland organoids.

The authors sincerely apologize for these oversights.

The original article^1^ has been updated.

## References

[1] Wu, Y. et al. Organoids in the oral and maxillofacial region: present and future. *Int. J. Oral Sci.* **16**, 61, 10.1038/s41368-024-00324-w (2024). 39482304 10.1038/s41368-024-00324-w PMC11528035

